# Supplementary material for: STREAM: A data-driven framework for physiological state monitoring in ICU patients
Source: PLOS Digit Health. 2026 Oct 1;5(10):e0001753. doi: 10.1371/journal.pdig.0001753 (PMC13630200; doi:10.1371/journal.pdig.0001753)
Supplement: Supplementary file 1 [file pdig.0001753.s001.docx]

**Supplementary Table 1.** Cohort characteristics of eICU-CRD and MIMIC-IV datasets

| **Characteristic** | **eICU-CRD (N=158,294)** | **MIMIC-IV (N=84,517)** |
| --- | --- | --- |
| **Demographics** |  |  |
| Age, years, mean (SD) | 63.2 (16.4) | 65.1 (17.2) |
| Male sex, n (%) | 85,992 (54.3%) | 47,854 (56.6%) |
| **ICU Characteristics** |  |  |
| ICU length of stay, days, median (IQR) | 1.9 (1.0–3.8) | 2.1 (1.2–4.2) |
| Hospital length of stay, days, median (IQR) | 5.8 (3.2–10.4) | 6.4 (3.5–11.8) |
| **Outcomes, n (%)** |  |  |
| ICU mortality | 8,474 (5.4%) | 5,867 (6.9%) |
| Hospital mortality | 14,119 (8.9%) | 8,870 (10.5%) |

*Abbreviations: SD, standard deviation; IQR, interquartile range; ICU, intensive care unit. Both cohorts included adult patients (≥18 years) with ICU stays exceeding 8 hours.*
