# Supplementary material for: STREAM: A data-driven framework for physiological state monitoring in ICU patients
Source: PLOS Digit Health. 2026 Oct 1;5(10):e0001753. doi: 10.1371/journal.pdig.0001753 (PMC13630200; doi:10.1371/journal.pdig.0001753)
Supplement: Supplementary file 3 [file pdig.0001753.s003.docx]

**S3 Table. Baseline model comparison and incremental value of state geometry features: DeLong test, integrated discrimination improvement, and net reclassification improvement in eICU-CRD and MIMIC-IV.**

**Part A: eICU-CRD (internal validation, n = 158,294)**

| **Time window** | **N patients** | **STREAM AUROC** | **Baseline AUROC** | **ΔAUROC** | **DeLong z** | **DeLong p** | **IDI (95% CI)** | **IDI p** | **NRI (95% CI)** | **NRI events** | **NRI non-events** | **NRI p** |
| --- | --- | --- | --- | --- | --- | --- | --- | --- | --- | --- | --- | --- |
| 8 hours | 127,765 | 0.863 | 0.795 | +0.067 | 28.430 | <0.001 | 0.130 (0.125-0.135) | <0.001 | 0.605 (0.582-0.628) | 0.261 | 0.344 | <0.001 |
| 24 hours | 152,824 | 0.873 | 0.810 | +0.063 | 28.857 | <0.001 | 0.115 (0.110-0.120) | <0.001 | 0.498 (0.477-0.518) | 0.295 | 0.203 | <0.001 |
| 48 hours | 156,927 | 0.890 | 0.832 | +0.058 | 28.093 | <0.001 | 0.127 (0.121-0.132) | <0.001 | 0.568 (0.546-0.589) | 0.269 | 0.299 | <0.001 |
| 72 hours | 157,788 | 0.903 | 0.848 | +0.055 | 28.508 | <0.001 | 0.124 (0.119-0.129) | <0.001 | 0.556 (0.536-0.577) | 0.285 | 0.272 | <0.001 |
| Full stay | 158,294 | 0.932 | 0.877 | +0.054 | 31.681 | <0.001 | 0.152 (0.146-0.157) | <0.001 | 0.699 (0.679-0.720) | 0.312 | 0.387 | <0.001 |

**Part B: MIMIC-IV (external validation, n = 84,517)**

| **Time window** | **N patients** | **STREAM AUROC** | **Baseline AUROC** | **ΔAUROC** | **DeLong z** | **DeLong p** | **IDI (95% CI)** | **IDI p** | **NRI (95% CI)** | **NRI events** | **NRI non-events** | **NRI p** |
| --- | --- | --- | --- | --- | --- | --- | --- | --- | --- | --- | --- | --- |
| 8 hours | 77,177 | 0.798 | 0.748 | +0.050 | 14.866 | <0.001 | 0.140 (0.134-0.145) | <0.001 | 0.326 (0.302-0.350) | −0.442 | 0.768 | <0.001 |
| 24 hours | 84,145 | 0.815 | 0.746 | +0.069 | 19.473 | <0.001 | 0.127 (0.122-0.133) | <0.001 | 0.380 (0.354-0.404) | −0.137 | 0.516 | <0.001 |
| 48 hours | 84,474 | 0.836 | 0.779 | +0.057 | 18.890 | <0.001 | 0.129 (0.123-0.134) | <0.001 | 0.382 (0.355-0.407) | −0.160 | 0.542 | <0.001 |
| 72 hours | 84,490 | 0.857 | 0.775 | +0.082 | 25.588 | <0.001 | 0.175 (0.169-0.181) | <0.001 | 0.480 (0.455-0.506) | −0.138 | 0.619 | <0.001 |
| Full stay | 84,517 | 0.899 | 0.799 | +0.100 | 32.281 | <0.001 | 0.274 (0.268-0.280) | <0.001 | 0.629 (0.603-0.655) | −0.102 | 0.731 | <0.001 |

*Incremental value of STREAM state geometry features over a baseline model using the same 26 clinical measurements and 2 demographics (age and gender) without state geometry features. DeLong tests compare correlated AUROCs from the same patients using the analytical method of DeLong et al. IDI (Integrated Discrimination Improvement) measures the average improvement in predicted probability separation between survivors and non-survivors. NRI (Net Reclassification Improvement) is the category-free NRI of Pencina et al. reporting the net proportion of patients correctly reclassified. In MIMIC-IV (Part B), the event-specific NRI component is negative, reflecting a cross-site calibration difference in absolute probability scale rather than a failure of discrimination; this pattern is consistent with known limitations of category-free NRI in external validation settings. The DeLong test and IDI, which are robust to calibration differences, consistently confirm STREAM's superiority in both cohorts. All p-values < 0.001. IDI, integrated discrimination improvement; NRI, net reclassification improvement; AUROC, area under the receiver operating characteristic curve; ΔAUROC, difference between STREAM and baseline AUROC.*
