# Supplementary material for: STREAM: A data-driven framework for physiological state monitoring in ICU patients
Source: PLOS Digit Health. 2026 Oct 1;5(10):e0001753. doi: 10.1371/journal.pdig.0001753 (PMC13630200; doi:10.1371/journal.pdig.0001753)
Supplement: Supplementary file 5 [file pdig.0001753.s005.docx]

**S5 Table. Data leakage prevention measures across the STREAM pipeline.**

| **Category** | **Potential leakage source** | **Prevention method** | **Implementation** | **Verification** |
| --- | --- | --- | --- | --- |
| **Temporal feature engineering** | Future-timepoint data entering early-horizon models | Time-restricted feature computation | • Each horizon model uses only its allowed intervals — 24 h: [pre-admission, 0–8 h, 8–16 h, 16–24 h]; 48 h adds 24–48 h; 72 h adds 48–72 h.  • Pre-computed values that span future timepoints are never used. | Filtered data confirmed to contain only the allowed intervals before any feature is calculated. |
| **Trajectory / importance features** | Importance features computed from future data | Time-filtered importance calculation | • Maintenance importance uses only the current and previous timepoints.  • Transition importance uses only the timepoints immediately before and after a transition. | Importance values confirmed to be computed from allowed timeframes only. |
| **Train / test split** | Information from the full dataset leaking into model training | Split-first methodology | • train_test_split() is performed BEFORE any imputation or scaling.  • Stratified by outcome; fixed random_state = 42 for reproducibility. | Train and test sets confirmed to be created before any preprocessing step. |
| **Missing-value imputation (model features)** | Test-set statistics influencing training | Training-only imputation statistics | • Medians (continuous) and modes (categorical) are computed from the training set only, then applied to both train and test.  • Imputation values stored for transparency. | Imputation statistics confirmed to be derived from training data before application to the test set. |
| **Class-imbalance handling** | Test-set outcome distribution affecting model weights | Training-only class weighting | scale_pos_weight is set from the training-set positive rate only. | Positive rate confirmed to be computed from the training labels, not the full cohort. |
| **Feature selection** | Outcome-related columns entering the feature matrix | Explicit exclusion lists | • Direct outcomes excluded (e.g., stay identifiers, expire flags, length-of-stay fields).  • Derived outcomes (length-of-stay extension, mortality) and metadata excluded.  • Experimental / ablation features not used in the final model are excluded. | Feature matrix checked against the maintained exclusion list. |
| **Severity-score & dataset-specific exclusions** | Embedded severity scores or identifiers acting as outcome proxies | Targeted exclusion | • APACHE and other precomputed severity-score columns dropped so the model learns physiology rather than precomputed risk.  • Dataset-native patient identifier used for splitting. | Severity-score and identifier columns confirmed removed before modeling. |
| **External validation (MIMIC-IV)** | Overfitting to the development dataset | Independent external validation (no development use) | • eICU-derived states, centroids, covariance, 95% boundaries, and trained models are applied to MIMIC-IV (n = 84,517) without retraining.  • MIMIC-IV measurements are harmonized to the eICU feature space prior to scoring. | • MIMIC-IV used only for external testing — never for development, tuning, or threshold selection.  • External performance is reported with and without distribution harmonization. |
| **Model-training pipeline order** | Inconsistent preprocessing order across model variants | Standardized pipeline order | (1) data loading and validation; (2) feature-type identification; (3) stratified train/test split; (4) training-only imputation; (5) training-only class weighting; (6) model training with early stopping; (7) evaluation on the held-out test set. | Pipeline order confirmed identical across all model variants. |
| **Evaluation methodology** | Optimistic performance estimation | Prespecified evaluation | • Operating thresholds chosen with Youden’s J statistic.  • Time-restricted metrics; bootstrap resampling (2,000 iterations) for confidence intervals. | Thresholds and metrics computed on held-out test data. |

*Summary of the measures used in STREAM to reduce the risk of data leakage and preserve temporal validity. State discovery is unsupervised and uses no outcome labels, so state centroids and covariance cannot transfer outcome information; the supervised mortality models use strict patient-level train/test separation, with imputation, class weighting, and threshold selection derived from training data only.*
