# Supplementary material for: STREAM: A data-driven framework for physiological state monitoring in ICU patients
Source: PLOS Digit Health. 2026 Oct 1;5(10):e0001753. doi: 10.1371/journal.pdig.0001753 (PMC13630200; doi:10.1371/journal.pdig.0001753)
Supplement: Supplementary file 6 [file pdig.0001753.s006.docx]

**S6 Table. External validation in MIMIC-IV without quantile-to-quantile distribution alignment.**

**Part A: eICU-derived threshold applied to MIMIC-IV (prospective deployment setting)**

| **Time window** | **AUROC (95% CI)** | **AUPRC** | **Threshold** | **Accuracy** | **Sensitivity** | **Specificity** | **PPV** | **NPV** | **LR+** | **LR-** |
| --- | --- | --- | --- | --- | --- | --- | --- | --- | --- | --- |
| 8 hours | 0.790 (0.788-0.792) | 0.331 | 0.453 | 0.466 | 0.895 | 0.432 | 0.111 | 0.981 | 1.58 | 0.24 |
| 24 hours | 0.812 (0.810-0.813) | 0.356 | 0.495 | 0.554 | 0.877 | 0.530 | 0.123 | 0.983 | 1.87 | 0.23 |
| 48 hours | 0.836 (0.835-0.838) | 0.395 | 0.426 | 0.566 | 0.898 | 0.542 | 0.128 | 0.986 | 1.96 | 0.19 |
| 72 hours | 0.857 (0.856-0.859) | 0.428 | 0.488 | 0.651 | 0.883 | 0.634 | 0.152 | 0.986 | 2.41 | 0.19 |
| Full stay | 0.899 (0.899-0.900) | 0.526 | 0.472 | 0.747 | 0.886 | 0.737 | 0.201 | 0.989 | 3.36 | 0.15 |

**Part B: MIMIC-IV-optimized threshold (site-recalibrated setting)**

| **Time window** | **AUROC (95% CI)** | **AUPRC** | **Threshold** | **Accuracy** | **Sensitivity** | **Specificity** | **PPV** | **NPV** | **LR+** | **LR-** |
| --- | --- | --- | --- | --- | --- | --- | --- | --- | --- | --- |
| 8 hours | 0.790 (0.788-0.792) | 0.331 | 0.671 | 0.754 | 0.675 | 0.760 | 0.182 | 0.967 | 2.81 | 0.43 |
| 24 hours | 0.812 (0.810-0.813) | 0.356 | 0.642 | 0.736 | 0.738 | 0.736 | 0.173 | 0.974 | 2.80 | 0.36 |
| 48 hours | 0.836 (0.835-0.838) | 0.395 | 0.609 | 0.758 | 0.764 | 0.758 | 0.190 | 0.977 | 3.15 | 0.31 |
| 72 hours | 0.857 (0.856-0.859) | 0.428 | 0.645 | 0.785 | 0.761 | 0.787 | 0.211 | 0.978 | 3.58 | 0.30 |
| Full stay | 0.899 (0.899-0.900) | 0.526 | 0.633 | 0.833 | 0.800 | 0.836 | 0.267 | 0.982 | 4.87 | 0.24 |

*Sensitivity analysis for external validation without quantile-to-quantile distribution matching. MIMIC-IV features were independently scaled using MIMIC's own parameters and corrected for unit and scaling differences using unit conversion and scaling only, without any distributional alignment. Part A applies the eICU-derived operating threshold directly (true prospective deployment). Part B shows performance at the MIMIC-IV-optimized threshold for reference. AUROC values differ by at most 0.008 from the q-q aligned Table 3 results (0.798, 0.815, 0.836, 0.857, 0.899), confirming that the quantile alignment did not artificially inflate performance. The high sensitivity and NPV in Part A reflect the eICU threshold, which is calibrated for high sensitivity; Part B shows the more balanced site-recalibrated operating point. AUROC, area under the receiver operating characteristic curve; AUPRC, area under the precision-recall curve; PPV, positive predictive value; NPV, negative predictive value; LR+, positive likelihood ratio; LR-, negative likelihood ratio.*
