# Supplementary material for: STREAM: A data-driven framework for physiological state monitoring in ICU patients
Source: PLOS Digit Health. 2026 Oct 1;5(10):e0001753. doi: 10.1371/journal.pdig.0001753 (PMC13630200; doi:10.1371/journal.pdig.0001753)
Supplement: Supplementary file 7 [file pdig.0001753.s007.docx]

**S7 Table. Mortality discrimination using optimal-transport versus classical change-point segmentation, with the Gaussian mixture state count for reference.**

| **Time window** | **AUROC (95% CI)** | **AUPRC** | **Sensitivity** | **Specificity** | **PPV** | **NPV** | **LR+** | **LR-** |
| --- | --- | --- | --- | --- | --- | --- | --- | --- |
| 8 hours | 0.828 (0.822-0.833) | 0.308 | 0.736 | 0.760 | 0.161 | 0.979 | 3.06 | 0.35 |
| 24 hours | 0.841 (0.834-0.846) | 0.340 | 0.716 | 0.803 | 0.174 | 0.980 | 3.65 | 0.35 |
| 48 hours | 0.858 (0.854-0.862) | 0.382 | 0.777 | 0.777 | 0.165 | 0.984 | 3.48 | 0.29 |
| 72 hours | 0.874 (0.869-0.880) | 0.417 | 0.787 | 0.797 | 0.181 | 0.985 | 3.89 | 0.27 |
| Full stay | 0.906 (0.905-0.908) | 0.481 | 0.825 | 0.831 | 0.216 | 0.988 | 4.87 | 0.21 |

*Performance of XGBoost trained on change-point-derived state geometry features (4 states: pre-admission, 0-8h, 8-24h, 24h to end), using breakpoints defined by the three largest inter-snapshot Euclidean distances. All XGBoost hyperparameters, feature engineering, and cross-validation procedures are identical to the published STREAM model. Metrics are reported at the Youden-optimal threshold. For comparison, STREAM (OT) achieved AUROC 0.863 to 0.932 across the same horizons (Table 2). GMM with BIC-optimal selection (3 states) was not separately evaluated by XGBoost because GMM operates in a 3-component PCA space that precludes computation of per-feature importance scores; the STREAM vs raw-feature baseline comparison (Supplementary Table S3) already quantifies the contribution of these scores to predictive performance. PPV, positive predictive value; NPV, negative predictive value; LR+, positive likelihood ratio; LR-, negative likelihood ratio.*
